# Genome-wide characterization of L-aspartate oxidase genes in wheat and their potential roles in the responses to wheat disease and abiotic stresses

**DOI:** 10.3389/fpls.2023.1210632

**Published:** 2023-07-05

**Authors:** Yanqun Feng, Mingshuang Tang, Junhui Xiang, Pingu Liu, Youning Wang, Wang Chen, Zhengwu Fang, Wenli Wang

**Affiliations:** ^1^ Ministry of Agriculture and Rural Affairs (MARA) Key Laboratory of Sustainable Crop Production in the Middle Reaches of the Yangtze River (Co-Construction by Ministry and Province)/Engineering Research Center of Ecology and Agricultural Use of Wetland, Ministry of Education, Hubei Collaborative Innovation Center for Grain Industry, College of Agriculture, Yangtze University, Jingzhou, China; ^2^ Nanchong Academy of Agriculture Sciences, Nanchong, Sichuan, China; ^3^ Hubei Key Laboratory of Quality Control of Characteristic Fruits and Vegetables, Hubei Engineering University, Xiaogan, Hubei, China; ^4^ College of Plant Protection, Northwest A&F University, Yangling, Shaanxi, China

**Keywords:** TaAO, gene structure, abiotic stresses, gene expression, quantitative PCR, biological functions

## Abstract

L-aspartate oxidase (AO) is the first enzyme in NAD^+^ biosynthesis and is widely distributed in plants, animals, and microorganisms. Recently, AO family members have been reported in several plants, including *Arabidopsis thaliana* and *Zea mays*. Research on AO in these plants has revealed that AO plays important roles in plant growth, development, and biotic stresses; however, the nature and functions of AO proteins in wheat are still unclear. In this study, nine *AO* genes were identified in the wheat genome *via* sequence alignment and conserved protein domain analysis. These nine wheat *AO* genes (*TaAOs*) were distributed on chromosomes 2, 5, and 6 of sub-genomes A, B, and D. Analysis of the phylogenetic relationships, conserved motifs, and gene structure showed that the nine *TaAOs* were clustered into three groups, and the *TaAOs* in each group had similar conserved motifs and gene structure. Meanwhile, the subcellular localization analysis of transient expression mediated by Agrobacterium tumetioniens indicated that TaAO3-6D was localized to chloroplasts. Prediction of cis-elements indicated that a large number of cis-elements involved in responses to ABA, SA, and antioxidants/electrophiles, as well as photoregulatory responses, were found in *TaAO* promoters, which suggests that the expression of *TaAOs* may be regulated by these factors. Finally, transcriptome and real-time PCR analysis showed that the expression of *TaAOs* belonging to Group III was strongly induced in wheat infected by *F. graminearum* during anthesis, while the expression of *TaAOs* belonging to Group I was heavily suppressed. Additionally, the inducible expression of *TaAOs* belonging to Group III during anthesis in wheat spikelets infected by *F. graminearum* was repressed by ABA. Finally, expression of almost all *TaAOs* was induced by exposure to cold treatment. These results indicate that TaAOs may participate in the response of wheat to *F. graminearum* infection and cold stress, and ABA may play a negative role in this process. This study lays a foundation for further investigation of *TaAO* genes and provides novel insights into their biological functions.

## 1 Introduction

L-aspartate oxidase (AO), a kind of flavin oxidase, converts aspartate to iminoaspartic acid using either molecular oxygen or fumarate as electron acceptors. It plays an indispensable role in the biosynthesis of nicotinamide adenine dinucleotide (NAD^+^) (Mattevi et al., 1999). NAD^+^ biosynthesis consists of five steps, of which the first is oxidation of L-aspartate into iminoaspartate catalyzed by L-aspartate oxidase (Katoh et al., 2006). NAD^+^ is an important component of the respiratory chain, so it plays an important role in biological energy metabolism. It additionally participates in reduction–oxidation reactions, DNA repair, ADP-ribosylation, and a series of metabolic processes (Gakiere et al., 2018). L-aspartate oxidase, as the enzyme of the first reaction in *de novo* synthesis of NAD^+^, is also thought to play an important role in the energy metabolism system and other metabolic pathways in organisms. Therefore, AO has been researched extensively over the years.

AO was initially reported in *Escherichia coli*, where the B protein of quinolinic synthase was identified as a L-aspartate oxidase (Nasu et al., 1982). Later, two conserved domains, the FAD binding domain and Succ_DH_flav_C, were found in AO proteins (Mattevi et al., 1999). Subsequently, AO was identified in *Pyrococcu shorikishii* OT-3 (Sakuraba et al., 2002), *Sulfolobus tokodaii* (Sakuraba et al., 2008), *Bacillus subtilis* (Marinoni et al., 2008), and *Pseudomonas putida* (Leese et al., 2013). Since then, the physiological and biochemical properties of AO have been extensively investigated in bacteria (Mortarino et al., 1996; Bifulco et al., 2013; Armenia et al., 2017; Chow et al., 2017). AO has the following two features: (a) *in vitro*, it is able to use different electron acceptors, such as oxygen, fumarate, cytochrome c, and quinones, suggesting that it is involved in NAD biosynthesis in anaerobic as well as aerobic conditions (Tedeschi et al., 1996); and (b) the primary and tertiary structures are not similar to those of other flavo-oxidases, but rather are similar to those of the flavoprotein subunit of the succinate dehydrogenase/fumarate reductase class of enzymes. AO can reduce fumarate and oxidize L-aspartate, but cannot oxidize succinate (Bacchella et al., 1999; Tedeschi et al., 2010). It has been reported that *Shigella*, a nicotinic acid auxotroph, is unable to synthesize NAD *via* the *de novo* pathway due to *AO* gene mutations. When AO function is restored in *Shigella*, sustained loss of virulence and inability to invade host cells are observed, which points toward AO as a locus of antivirulence (Prunier et al., 2007).

Compared with microorganisms, little is still known about AO in plants. Up to this point, AO proteins have been reported only in maize and *A. thaliana*. In maize, a gene *GRMZM2G139689* has been reported to encode AO protein. At the mononuclear stage of microspore development, the expression level of this gene was found to be greatly downregulated in male sterile line C48-2 compared with maintainer line 48-2 (Dong, 2019). This suggests that AO protein may be involved in pollen abortion in maize. In *A. thaliana*, *At5g14760* has been identified as an *AO* (Macho et al., 2012). Overexpression of *AtAO* increases NAD^+^ content, and loss of AtAO activity results in a decrease in NAD^+^ levels (Hao et al., 2018). It is worth noting that the expression of *AOs* is upregulated in *A. thaliana* leaves infected by avirulent *Pseudomonas syringae* pv. *tomato* strain (Petriacq et al., 2012). Furthermore, research with an *AO A. thaliana* mutant has shown that AO is required for reactive oxygen species (ROS) bursts triggered by pathogen-associated molecular patterns and for stomatal immunity (Macho et al., 2012). These studies indicate that *AO* genes play important roles in regulating plant development and response to biotic stresses.

Wheat is one of the top three crops worldwide (Guo et al., 2018). Almost 60% of the wheat produced globally is consumed as food (Sobolewska et al., 2020), and global demand for wheat is expected to grow by approximately 70% over the next 30 years with growing populations, rising income levels, and increasing household consumption (Abedi and Mojiri, 2020). Wheat often suffers from exposure to biotic and abiotic stresses during growth; it is unclear whether wheat AO participates in the plant’s response to biotic/abiotic stresses, and the molecular characteristics of wheat AO are also unclear. In this study, TaAO genes were identified *via* sequence alignment and protein domain analysis, and the gene structure, phylogenetic relationships, and chromosome distribution of *TaAO* family genes were subsequently analyzed systematically using bioinformatics methods. Finally, the expression patterns of *TaAO* family genes were quantified *via* transcriptome analysis and qRT-PCR. This study lays a foundation for further analysis of *AO* genes in wheat.

## 2 Materials and methods

### 2.1 Genome-wide identification of *AO* genes in *T. aestivum*, *Ae. tauschii*, *T. urartu*, and *T. dicoccoides*

Genome data for *T*. *aestivum* (IWGSCv2.1), *Ae*. *tauschii* (v4.0.43), *T*. *urartu* (v1.43), and *T*. *dicoccum* (v1.0.43) were downloaded from Ensembl Plants database (http://plants.ensembl:index.html). Hidden Markov models (HMMs) for the FAD binding domain (PF00890.27) and the Succ_DH_flav_C domain (PF02910.23), obtained from the Pfam database (http://pfam.xfam), were used as queries to identify wheat AOs using HMMER3.0 (http://hmmer:download.html) with hit sequences specified as those with an e-value below 1e^-5^. Three *Arabidopsis* AOs (AtAOs), three maize AOs (ZmAOs), and two rice AOs (OsAOs), where the identification method was similar to that of *TaAOs*, were retrieved from genome databases for *Arabidopsis* (http://www.arabidopsis:index.jsp), maize (https://www.maizegdb.org), and rice (http://rice.plantbiology.msu.edu), respectively (**Table S1**). These AO proteins were used as queries to search for AO proteins in the genomes of *T. aestivum, Ae. tauschii, T. urartu*, and *T. dicoccoides via* BLASTp. Hit sequences with an e-value below 1e^-5^ were retained. The wheat AO candidates obtained using the above two methods were combined, and the non-redundant proteins were further analyzed using Pfam (v31.05) (http://pfam.sanger.ac.uk/search) and SMART (http://smart.embl-heidelberg.de/) (Letunic and Bork, 2018). Only these proteins that contained both the FAD binding domain and the Succ_DH_flav_C domain were considered to be wheat AOs. The *AO* genes were named according to their distribution on the chromosomes.

### 2.2 Characteristics of TaAO proteins

The protein sequence length, isoelectric point (pI), molecular weight (MW), instability index, and grand average of hydropathicity (GRAVY) of TaAOs were predicted using the ExPASy online tool (https://www.expasy.org/) (Gasteiger et al., 2003). Subcellular localization of TaAOs was predicted using the WoLF PSORT online tool (https://wolfpsort.hgc.jp/) (Chou and Shen, 2010).

### 2.3 Phylogenetic analysis of TaAOs

The sequences of AO proteins from *A. thaliana*, rice, maize, *T. aestivum*, *Ae. tauschii*, *T. urartu*, and *T. dicoccoides* were aligned using the ClustalW2 software package (Thompson et al., 1994). A neighbor-joining (NJ) phylogenetic tree was constructed using the MEGA X software package (Mega Limited, Auckland, New Zealand) (Kumar et al., 2018) with 1000 bootstrap repetitions. Finally, the tree was modified using the Interactive Tree of Life tool (iTOL, v6, http://itol.embl.de) (Letunic and Bork, 2021).

### 2.4 Genomic organization of TaAOs in wheat

Information on the position of TaAOs in wheat chromosomes was extracted from annotated information on the wheat genome. The physical map was drawn using the MapInspect software package. Information on the exon–intron structure of *TaAO* genes was visualized using the TBtools software package (Chen et al., 2020). Conserved motifs of TaAOs were identified using the MEME suite, with the following parameter settings: number of motifs, up to 15; width range, from 6 to 50 amino acids. The outputs on the motif structures of TaAO proteins were displayed using TBtools. Gene duplication events were analyzed according to the method described by Panchy et al. (2016) and illustrated using the Circos package in TBtools. For further examination of the footprints of selection during the processes of domestication (wild emmer and *Ae. tauschii* versus landraces) and improvement (landraces versus varieties), we overlapped the identified *AO* genes with the sweep region identified by Cheng et al. (2019) to check whether they were selected. The Ka and Ks values and the Ka/Ks ratio were calculated using TBtools. A Ka/Ks value of 1 indicates a neutral selection effect; a Ka/Ks value >1 indicates positive selection for evolutionary acceleration; and Ka/Ks <1 indicates purifying selection under function constraints.

### 2.5 RNA isolation and cDNA first-strand synthesis

Total RNA was extracted using TRIzol reagent (Invitrogen, USA) according to the manufacturer’s instructions. Subsequently, 1 μg RNA was used for cDNA first-strand synthesis using a PrimeScript RT reagent kit with gDNA Eraser (Takara, China) according to the manufacturer’s instruction.

### 2.6 Subcellular localization analysis of TaAO3-6D protein

Subcellular localization analysis of a TaAO protein was performed using an *Agrobacterium tumefaciens* mediated transient expression system in leaves of *Nicotiana benthamiana.* First, the coding sequence of a TaAO, *TaAO3-6D*, was amplified (the primers are listed in **Table S2**) and cloned into a GFP fusion protein expression vector pCAMBIA1300-GFP. Next, the recombinant vector was transformed into an *Agrobacterium tumefaciens* GV3101 strain. Positive clones were cultured and injected into the leaves of 5- to 6-week-old *Nicotiana benthamiana.* These were observed using a fluorescence microscope (Olympus FV3000, Tokyo, Japan) 48 h after injection.

### 2.7 Cis-acting element and protein interaction network analyses of TaAOs

The 1.5 kb sequence upstream of the start codon of each of the *TaAO* genes was obtained, and these sequences were used for the prediction of cis-acting elements *via* the PlantCARE website (http://bioinformatics.psb.ugent.be/webto-ols/plantcare/html/). The cis-acting elements were arranged and displayed using the R software package “pheatmap” (Rombauts et al., 1999). To study the protein–protein interactions (PPIs) between TaAOs and other proteins, a protein network was generated using the STRING v11.5 webserver (https://cn.string-db.org/).

### 2.8 Expression profiling of *TaAO* genes *via* transcriptome analysis

Transcriptome data on sequences involved in wheat growth and wheat responses to biotic stresses (*Fusarium graminearum*, stripe rust, and wheat powdery mildew) and abiotic stresses (phosphorous starvation, cold, heat, and drought) were downloaded from the NCBI database (**Table S3**) and mapped to the wheat reference genome *via* Hisat2 (Fang et al., 2020). The expression levels of *TaAOs* were calculated using Cufflinks (Trapnell et al., 2012). All transcript values were standardized by log2 (TPM + 1) transformation, and the expression profiles of *TaAOs* were generated using the R package “pheatmap”.

### 2.9 Growth and stress treatments of wheat seedlings

Jingshuang 16, a wheat cultivar moderately susceptible to powdery mildew and rust (Ren et al., 2015), was used in this study. The wheat seeds were disinfected with 1% hydrogen peroxide; subsequently, after washing with distilled water, the seeds were kept at 25°C for 2 days for germination. The seedlings were cultured in quarter-strength Hoagland nutrient solution for 3 days, and then transferred to one-half Hoagland nutrient solution (pH=6.0) (Bishop and Bugbee, 1998). To examine the differential expression of *TaAOs* in response to drought and abscisic acid (ABA) treatment, seedlings were cultured in a greenhouse at 25/20°C under a 16 h light/8 h dark cycle. The seedlings were treated with 20% PEG6000, once at the heart stage and once at the leaf stage. Wheat leaves were sampled at 0, 2, 12, 24, 36, and 48 h after treatment. For the ABA treatment, ABA was added to one-half strength Hoagland nutrient solution at a final concentration of 100 μM. After 0, 2, 6, 12, 24, and 48 h of treatment, leaves were harvested for further research. Finally, for the stripe rust infection treatment, seedlings were cultured in a plant growth chamber under a 16 h light/8 h dark cycle at 16°C. Wheat leaves were inoculated with fresh uredospores of stripe rust CYR32 using the smearing method and kept in dark, moist conditions for 24 hours to promote infection (Santra et al., 2008). Leaves were collected at 0, 6, 12, 24, and 48 h. All samples were immediately frozen in liquid nitrogen and stored at -80°C for future use.

### 2.10 qRT-PCR analysis

Real-time PCR reaction systems were used to carry out reaction schemes following the manufacturer’s instruction (Vazyme, China). Gene-specific primers (**Table S4**) were designed using the Primer 5.0 software package. The ADP-ribosylation factor *Ta2291* was used as the internal reference gene for qRT-PCR analysis. Each experiment was carried out with three biological replicates, and three technical repeats were performed for each replicate.

## 3 Results

### 3.1 Identification and classification of *AO* genes in wheat

**Figure 1:**
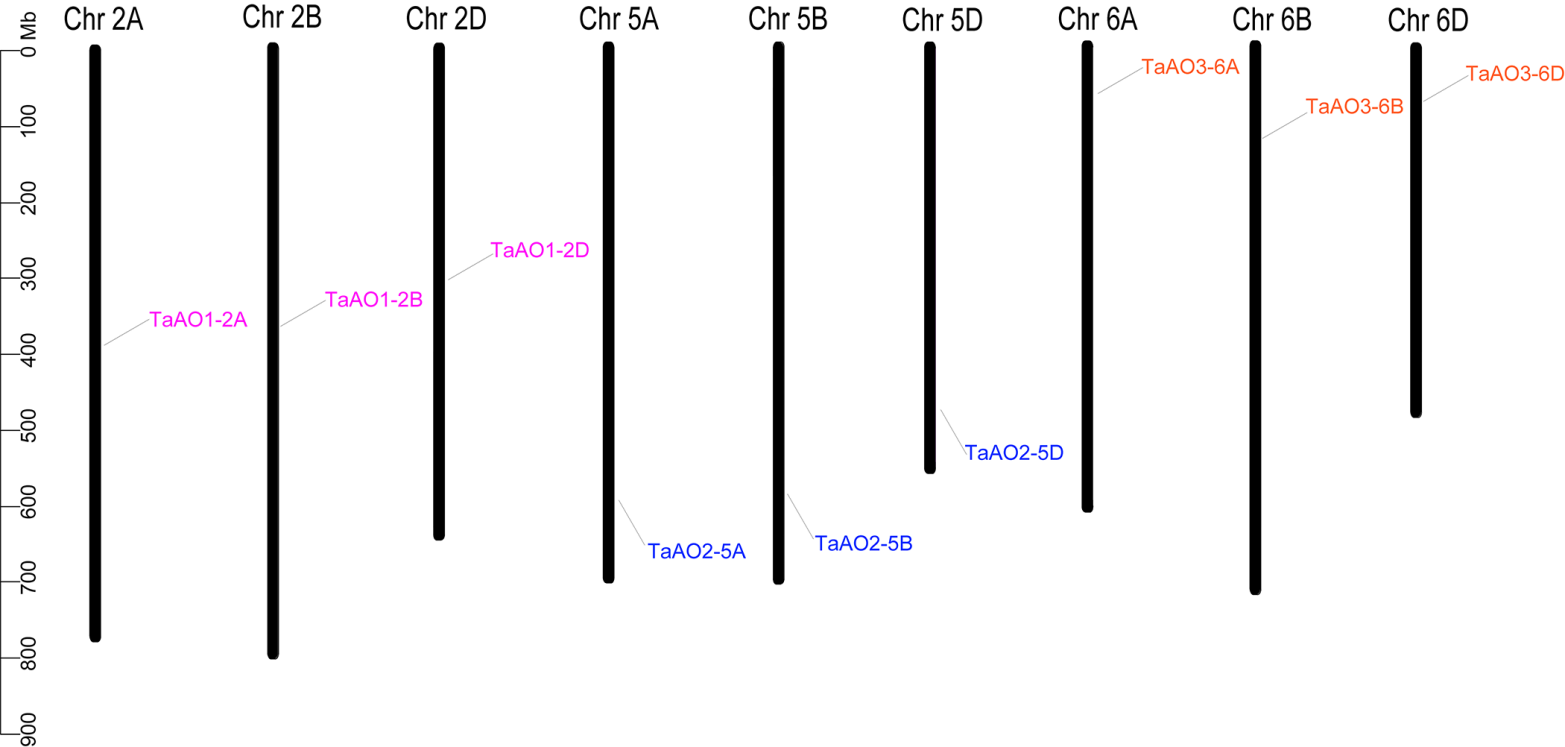
Chromosome locations of the nine TaAOs in wheat. The ruler on the left represents the physical distance between genes (Mbp).

Ten AO candidates were obtained from the wheat genome *via* HMM search. Meanwhile, the same ten AO candidates were retrieved from the wheat genome *via* BLASTp. Of these, one gene without the Succ_DH_flav_C domain was excluded, and the remaining nine candidate genes containing both of the FAD binding domain and the Succ_DH_flav_C domain were identified as *TaAOs* (**Table 1**; **Table S5**). Using the same procedure, four, three, and six *AOs* were identified for *T. urartu*, *Ae. Tauschii*, and *T. dicoccoides*, respectively (**Table S4**). The locations of *AO* genes on the wheat chromosomes were determined and analyzed for genomic homology; this analysis indicated that nine *TaAO* genes were distributed evenly on chromosomes 2, 5, and 6 of sub-genomes A, B, and D, while no tandem duplication or segmental duplication events were found (**Figure 1**).

**Table 1:** Protein features of AOs in *Triticum aestivum*.

Name	Locus ID	Len	MW	pI	II	Stability	GRAVY	Sub
TaAO1-2A	TraesCS2A03G0615500.1	619	68045.78	6.22	34.07	stable	-0.348	mitochondrion
TaAO1-2B	TraesCS2B03G0694000.1	571	62522.66	6.3	30.66	stable	-0.295	mitochondrion
TaAO1-2D	TraesCS2D03G0589200.1	619	67956.76	6.3	32.97	stable	-0.347	mitochondrion
TaAO2-5A	TraesCS5A03G0972600.1	621	68317.15	6.11	33.17	stable	-0.361	mitochondrion
TaAO2-5B	TraesCS5B03G1021700.1	621	68289.1	6.11	33.31	stable	-0.362	mitochondrion
TaAO2-5D	TraesCS5D03G0925000.1	591	65208.54	5.98	33.98	stable	-0.393	mitochondrion
TaAO3-6A	TraesCS6A03G0227300.1	641	70704.89	6.79	39.48	stable	-0.186	chloroplast
TaAO3-6B	TraesCS6B03G0316000.1	641	70780.01	6.94	38.80	stable	-0.206	chloroplast
TaAO3-6D	TraesCS6D03G0185200.1	641	70639.88	6.94	39.10	stable	-0.194	chloroplast

Len, amino acid length (aa); MW, molecular weight (KDa); pI, isoelectric point; II, instability index; GRAVY, grand average of hydropathy; Sub, subcellular localization.

### 3.2 Analysis of TaAO protein characteristics

**Figure 2:**
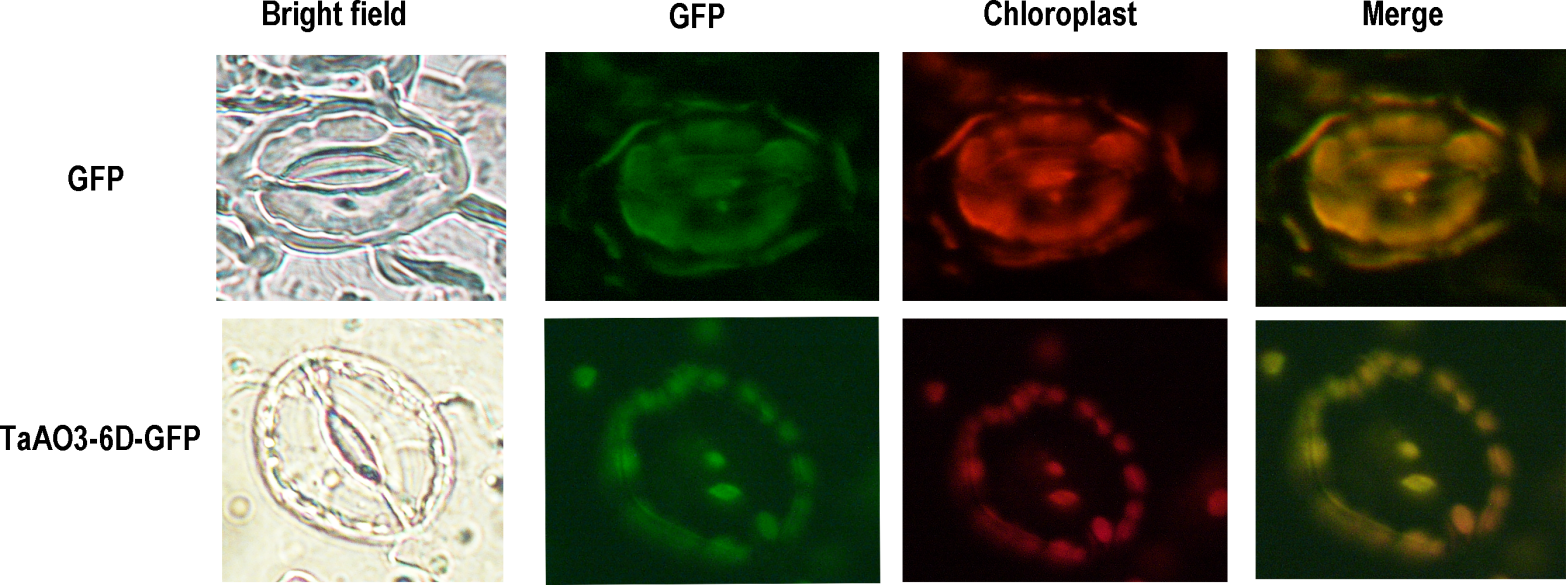
GFP-fused proteins of TaAO3-6D were localized to the chloroplasts of *Arabidopsis* guard cells. Chloroplast autofluorescence (red) and GFP fluorescence (green) in guard cells were simultaneously observed. Bright-field images (on the left) show the shapes of guard cells. Unfused GFP was found in the cytoplasm and nucleus, whereas TaAO3-6D-GFP was found in the chloroplasts. Scale bar = 10 μm.

To further understand the characteristics of the TaAO proteins, protein length, molecular weight, instability index, isoelectric points, average hydrophilicity coefficients, and predicted subcellular localization were analyzed. As shown in **Table 1**, the protein length of the TaAOs ranged from 571 to 641 aa, and molecular weight ranged from 62.5 to 70.7 kDa. Instability index ranged from 30.66 to 39.48, indicating that these TaAOs were all stable proteins (instability index < 40). The isoelectric points of these TaAOs fell between 5.98 and 6.94, which showed that they were acidic proteins. Their average hydrophilicity coefficients ranged from 0.186 to 0.393, indicating that they were hydrophilic proteins. Subcellular localization prediction *via* the WoLF PSORT software package indicated that TaAO3-6A, TaAO3-6B, and TaAO3-6D were localized to chloroplasts, while TaAO1-2A, TaAO1-2B, TaAO3-3D, TaAO2-5A, TaAO2-5B, and TaAO2-5D were localized to mitochondria. The results of TaAO3-6D-GFP fusion protein expression assays showed that TaAO3-6D was localized to chloroplasts (**Figure 2**), which was consistent with the predicted results.

### 3.3 Conserved motifs and gene structures of *TaAOs*

**Figure 3:**
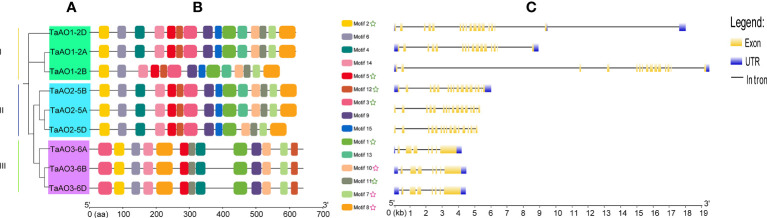
Phylogenetic analysis, conserved motifs, and gene structure of TaAOs. **(A)** Phylogenetic tree of TaAOs. The tree was built using the neighbor-joining (NJ) method with 1000 bootstrap repetitions in MEGA7. **(B)** Motifs of TaAO identified using MEME; MAST was used to visualize the patterns. Each motif is indicated with a specific color. The green star symbol represents the FAD binding domain; the purple star symbol represents the Succ_DH_flav_C domain. **(C)** Exon–intron structure of TaAOs, analyzed using GSDS. Untranslated regions (UTRs) are represented by blue frames; exons are represented by yellow frames; and introns are represented by black lines.

To understand the evolutionary relationships between TaAOs, a phylogenetic tree was constructed. As shown in **Figure 3A**, three *TaAOs* located on chromosome 2 were grouped into Group I, three *TaAOs* located on chromosome 5 were grouped into Group II, and the three genes located on chromosome 6 were clustered into Group III. The results of a conserved motifs analysis of the TaAOs (**Figure 3B**; **Table S6**) showed that all TaAO proteins contained Motifs 1, 2, 3, 5, 7, 8, 10, 11, and 12. Motifs 7, 8, and 10 were contained in the Succ_DH_flav_C domain and Motifs 1, 2, 3, 5, 11, and 12 were contained in the FAD binding domain. The number and order of motifs in TaAOs belonging to Groups I and II were essentially consistent, with the exceptions of *TaAO1-2B* lacking Motif 4 and *TaAO2-5D* lacking Motif 13. The number of motifs of Group III members differed from that of members of Groups I and II. TaAOs in Group III only had 13 motifs, from which Motifs 13 and 15 were absent. Furthermore, the order of motifs in Group III also differed from that of the other two groups. Additionally, the results on intron/exon distribution patterns of *TaAO* genes appeared to indicate that the number of exons was significantly greater in Groups I and II than in Group III. Generally, *TaAOs* belonging to Groups I and II contained 16 exons, with the exception of *TaAO1-2D*, which only contained 15 exons. In contrast, the *TaAOs* belonging to Group III only contained seven exons (**Figure 3C**).

### 3.4 Phylogenetic and Ka/Ks analysis of *AO* genes of *T. aestivum* and its ancestor species

**Figure 4:**
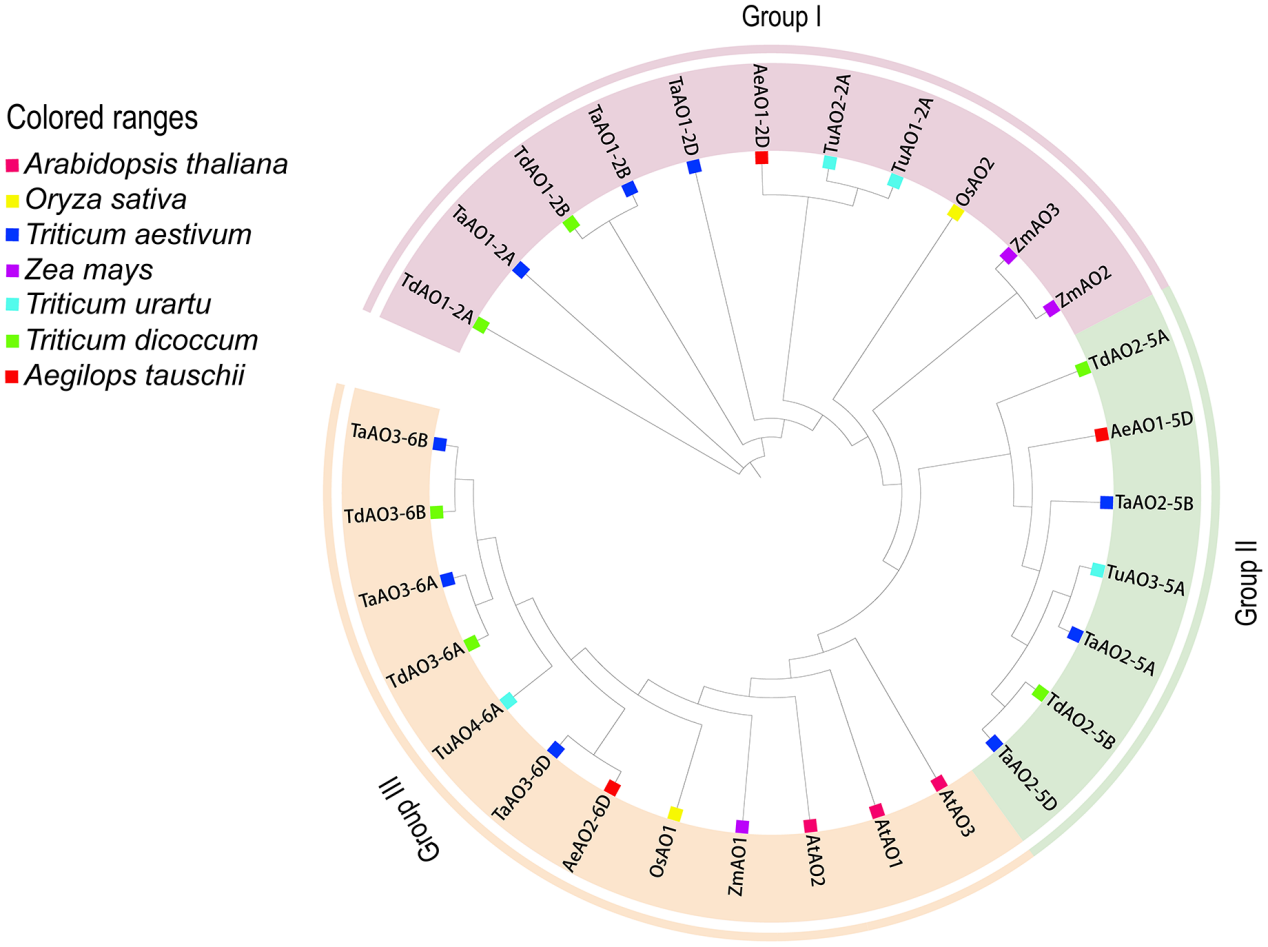
Phylogenetic tree of AOs from wheat, maize, rice, *Arabidopsis*, *T. urartu*, *T. dicoccoides*, and *Ae. tauschii*. The protein sequences were aligned using ClustalW2, and the phylogenetic tree was constructed using neighbor-joining (NJ) (1000 repeats) in the MEGA 7.0 software package. The AOs of wheat, maize, rice, *Arabidopsis*, *T. urartu*, *T. dicoccoides*, and *Ae. tauschii* can be distinguished by the use of different colors and shapes.

To further evaluate the phylogenetic relationships of TaAOs with other plant AOs, nine AOs from *T. aestivum*, four from *T. urartu*, three from *Ae. Tauschii*, six from *T. dicoccoides*, two from *Oryza sativa*, three from *Zea mays*, and three from *A. thaliana* were used to construct a phylogenetic tree. As shown in **Figure 4**, the set of all AOs could be divided into three groups, which was consistent with the above-described results on the phylogenetic tree of wheat AOs. TaAOs located on chromosome 2 in different sub-genomes were classified into Group I, TaAOs located on chromosome 5 were in Group II, and TaAOs on chromosome 6 were in Group III (**Table S7**). It is interesting that all three AtAOs were clustered into Group III, which was found to have the closest relationship with TaAOs on chromosome 6. The AOs of rice and *Zea mays* were classified into Groups I and III, with none falling into Group II. With regard to sub-genome donor species, members were distributed across the three groups. These results indicate that AOs in these species may have evolved under different evolutionary directions, and furthermore, the functions of AOs in different groups may be at variance with one another.

**Figure 5:**
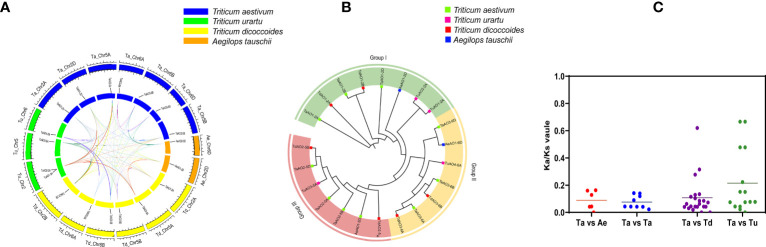
Analysis of **(A)** synteny and **(B)** phylogeny for *AO* genes in *T. aestivum* and its sub-genomic progenitors *T. urartu*, *T. dicoccoides*, and *Ae. Tauschii.* **(A)** Orange rectangles represent *Ae. tauschii* chromosomes, green rectangles represent *T. urartu* chromosomes, blue rectangles represent *T. aestivum* chromosomes, and yellow rectangles represent *T. dicoccoides* chromosomes. **(B)** This phylogenetic tree was constructed using 1000 bootstrap repetitions under the neighbor-joining (NJ) method in MEGA7. Blue, red, purple, and green squares represent *Ae. Tauschii*, *T. dicoccoides*, *T. urartu*, and *T. aestivum*, respectively. **(C)** Ka/Ks values for AO orthologous gene pairs between *T. aestivum*, *T. urartu*, *T. dicoccoides*, and *Ae. Tauschii*.

A homology analysis of wheat and three sub-genome donor species was conducted, and the orthologs and paralogs were clustered. Orthologs are defined as genes in different species that are derived from a single gene in the last common ancestor, and paralogs are homologous genes within a single species, resulting from gene duplication (Remm et al., 2001). A total of 53 homologous gene pairs were associated with *T. aestivum* (**Figure 5A**), of which nine were paralog gene pairs and 44 were ortholog gene pairs were found. Among these 44 ortholog gene pairs, there were 7, 22, and 15 ortholog genes between *T. aestivum* and each of its ancestor species (*Ae. Tauschii*, *T. dicoccoides*, and *T. Urartu*, respectively). *TaAO* genes the AOs of *Ae. tauschii*, *T. dicoccoides*, *T. urartu* and *T. aestivum* can be divided into three groups (**Figure 5B**) were not found in either the domestication-related or the improvement-related sweep regions, and all the Ka/Ks values for AO replication gene pairs were <1 (**Figure 5C**; **Table S8**), indicating that *TaAO* genes were purified and selected, and their functions may be conserved.

### 3.5 Cis-element analysis of *TaAO* genes

**Figure 6:**
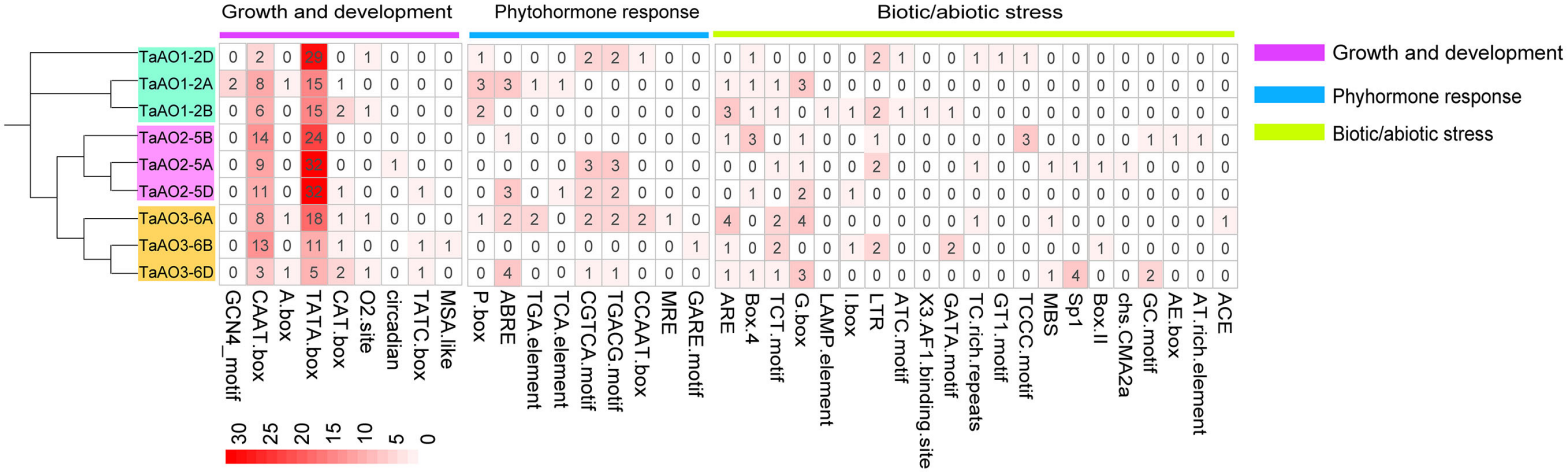
Identification of cis-acting elements of *TaAO* genes. The color gradient in each grid represents the number of promoter elements of these *AO* genes.

In the process of plant growth and development, not only can cis-regulatory elements regulate the spatio-temporal expression of genes, but they also are involved in responses to phytohormone exposure and abiotic stresses (Davis, 2009). In the present study, 39 kinds of cis-elements were identified in the promoter regions of *AO* genes in wheat (**Figure 6**; **Table S9**). Cis-elements involved in growth and development, including the TATA box and CAAT box, were found in all TaAO promoters. For cis-elements associated with plant hormone responses, the largest set of cis-elements was that of abscisic acid response elements (ABRE), of which 13 were identified. The second largest sets of cis-elements were salicylic acid-associated elements (CGTCA motif and TCA elements); the number of elements identified for each of these was 10. This suggests that the expression of *TaAOs* may be regulated by ABA and SA. The TGACG motif (methyl jasmonate), TGA element (auxin), CGTCA motif, and TCA element (salicylic acid) were identified in most of the *TaAO* gene promoters. In terms of biotic and abiotic stresses, the most abundant cis-element was the G-box, with 14 elements identified, followed by ARE, with 11. This suggests that the expression of *TaAOs* may be regulated by antioxidant/electrophile and photoregulatory factors. In summary, in addition to biotic stress regulation of AO, AO may also be regulated by ABA and SA hormones, as well as antioxidant/electrophile and photoregulatory abiotic stresses.

### 3.6 Molecular interaction networks

**Figure 7:**
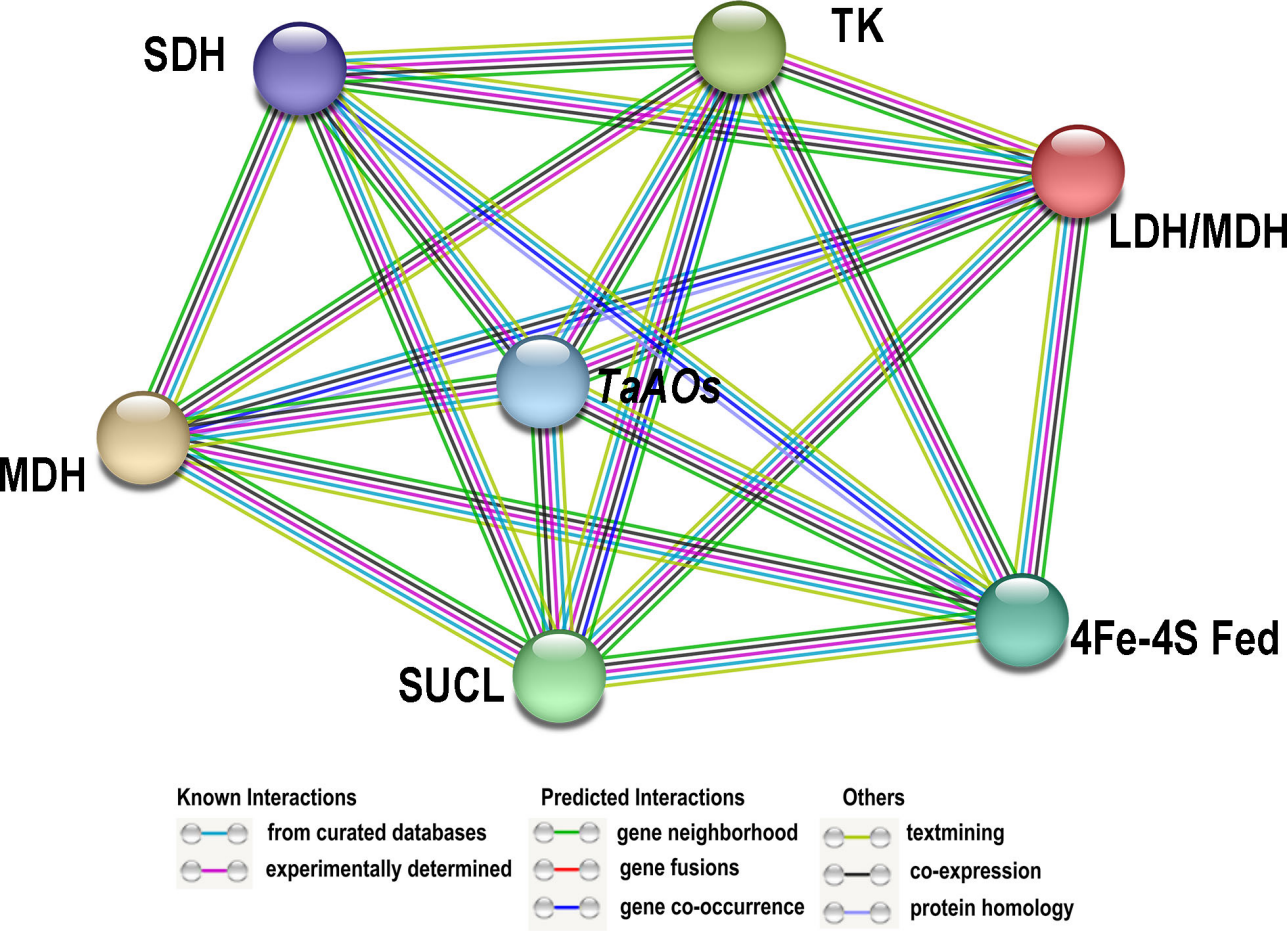
Protein–protein interaction (PPI) analysis of TaAO proteins. PPI network generated using STRINGV 11.5. Each node denotes a protein, and each edge represents an interaction.

A network of the interactions between TaAOs and other wheat proteins was built using STRING v11.0. The results showed that all nine TaAO proteins interacted with 17 wheat proteins. Among these 17 wheat proteins, seven (Traes_5BL_402FB4A3F.1, Traes_7AL_C0609756B.1, Traes_7BL_8F49CE9D6.2, Traes_2AS_4AB51ACE2.1, Traes_2AS_BA55E613D.2, Traes_7AL_6405AB56F.2, Traes_4AS_2DCA42965.1) were unknown proteins, and the remaining ten were Succinate-CoA ligases (Traes_2DS_3AC11B9D8.1, Traes_2AS_3BA916807.1, Traes_2BS_8863D42E7.1), 4Fe-4S ferredoxin-type protein (Traes_5DL_885A58CBA.3), lactate/malate dehydrogenase (Traes_1BL_A93F9F079.2, Traes_1DL_5A31A68D3.2, Traes_1AL_2EC98608D.2), malate dehydrogenase (Traes_1BL_BD3E22844.1), succinate dehydrogenase (Traes_7DL_91E866851), and transket_pyr protein (Traes_2AL_1E2B26B7F.1) (**Figure 7**; **Table S10**). Succinate-CoA ligase (SUCL) can promote the production of ATP during the conversion of malate to succinate in the TCA cycle (Ostergaard, 2008). 4Fe-4S ferredoxin-type protein (4Fe-4S Fed) is involved in various redox processes in organisms, such as DNA repair, RNA and protein modification, and cofactor synthesis (Feng et al., 2021). Lactate/malate dehydrogenases (LDH/MDH) are involved in energy metabolism. Lactate dehydrogenase (LDH) operates at the final stage of aerobic glycolysis. Malate dehydrogenase (MDH) is a key enzyme in the regulation of malate metabolism; it catalyzes the reversible oxidative decarboxylation of malate to produce pyruvate and CO_2_, as well as the reduction of NAD(P)^+^ (Zhao et al., 2022). Succinate dehydrogenase (SDH) is the only enzyme that participates in both the tricarboxylic acid or citric acid cycle and the electron transport chain (Gill, 2012). Transket_pyr protein (TK) plays an important role in carbon metabolism (Liu et al., 2023). These AO-interacting proteins all play important functions in energy metabolism, which explains why AO also plays such an important role in energy metabolism. These results provide clues for further study of the function of *TaAO* genes.

### 3.7 Transcriptome analysis of *TaAOs*

**Figure 8:**
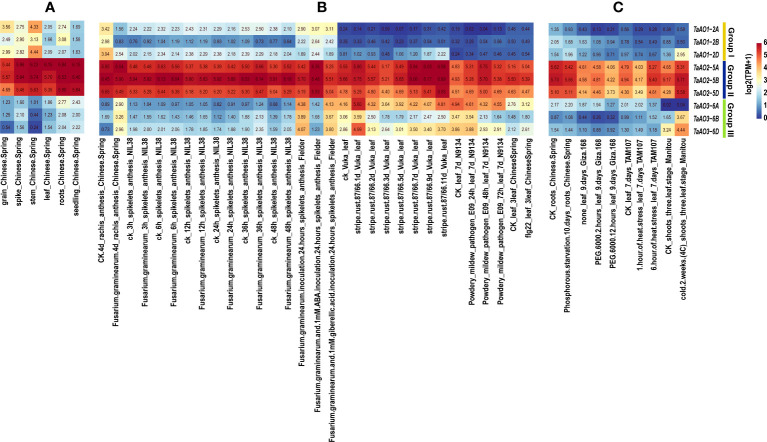
Transcriptome analysis of TaAOs. **(A)** Expression levels of nine *TaAO* genes in different tissues of Chinese Spring wheat. **(B)** Gene expression profiles of *TaAOs* under exposure to biological stress, plant hormone treatment, and pathogen-associated molecular patterns (PAMP). **(C)** Expression patterns of *TaAO* genes in wheat plants under exposure to different abiotic stresses. Different colors indicate the gene expression level: red indicates higher levels of expression, and blue lower.

In order to understand the expression patterns of *TaAOs*, wheat transcriptome data taken from different tissues and under exposure to different abiotic and biotic stresses were analyzed. From **Figure 8**, it is clearly evident that the expression levels of *TaAOs* belonging to Group II were the highest in all selected transcriptomes, which indicates that these three TaAOs may play essential roles in plant growth and response to various treatments. In terms of the different tissues, *TaAOs* belonging to Group I were relatively highly expressed in the spike, grain, stem, and root, and *TaAOs* belonging to Group III were relatively highly expressed in the root and seedling, while *TaAOs* belonging to Group II were highly expressed in all tissues, with no clear tissue specificity observed (**Figure 8A**).

As shown in **Figure 8B**, the transcriptomes of TaAOs under biotic stress were analyzed. In general, the expression of *TaAOs* changed slightly after infection by stripe rust. With the exceptions of *TaAO1-2A* and *TaAO3-6B*, the expression of *TaAOs* was induced by this form of stress. Interestingly, expression of *TaAO1-2D* was induced to a large extent at the late stage of infection (9 d and 11 d), while expression of *TaAO3-6D* was significantly induced at the early stage of infection (1d). These findings suggest that *TaAOs* might play a role in the response to stripe rust infection, but the patterns of expression were different for different *TaAO*s. In wheat N9134 from leaf tissue infected by powdery mildew, the expression levels of *TaAO*s also changed slightly. The expression of *TaAO1-2A* and of TaAOs belonging to Group III was suppressed, while the expression of other *TaAOs* was induced. The expression of *TaAOs* was also slightly altered in wheat NIL38 from spikelet tissue infected by *F. graminearum*, but it was greatly altered in *F. graminearum*-infected rachis tissue of Chinese Spring. In rachis tissue infected by *F. graminearum*, the expression of *TaAOs* belonging to Group I was repressed. The *TaAOs* with the greatest decrease in expression were *TaAO1-2B*, the expression levels of which decreased to 11.2% of the levels observed in CK, while the expression of *TaAOs* belonging to Group III was induced. The *TaAOs* with the greatest increase in expression were *TaAO3-6D*, the expression level of which was 10.33 times that observed in CK. These findings indicate that TaAOs may be involved in the pathogenesis of Fusarium head blight, and that TaAOs belonging to Groups I and III play different roles. Additionally, we found that the expression levels of *TaAOs* belonging to Group III were much lower in *F. graminearum*-infected wheat spikelet tissue treated with 1 mM ABA than in non-ABA-treated wheat spikelet tissue. This indicates that ABA negatively regulates the expression of *TaAOs* belonging to Group III in *F. graminearum*-infected wheat spikelets.

As shown in **Figure 8C**, the transcriptomes of TaAOs under abiotic stress were also analyzed. The results showed that the most significant changes in expression levels of *TaAOs* were found in wheat in response to cold treatment. The expression of all *TaAOs* was induced, with the exception of *TaAO1-2B*, the expression level of which was decreased by half. In contrast, the expression of most *TaAOs* was repressed during exposure to heat stress and drought stress. These results indicate that most TaAOs may participate in the response to cold and that they play different roles in the responses to heat and drought.

### 3.8 Quantitative real-time PCR analysis

To further understand the potential role of *TaAO* genes in biotic and abiotic stresses, the patterns of expression of *TaAO1-2D*, *TaAO2-5A*, and *TaAO3-6D* in response to stripe rust infection, ABA, and PEG stress were quantified *via* qRT-PCR.

**Figure 9:**
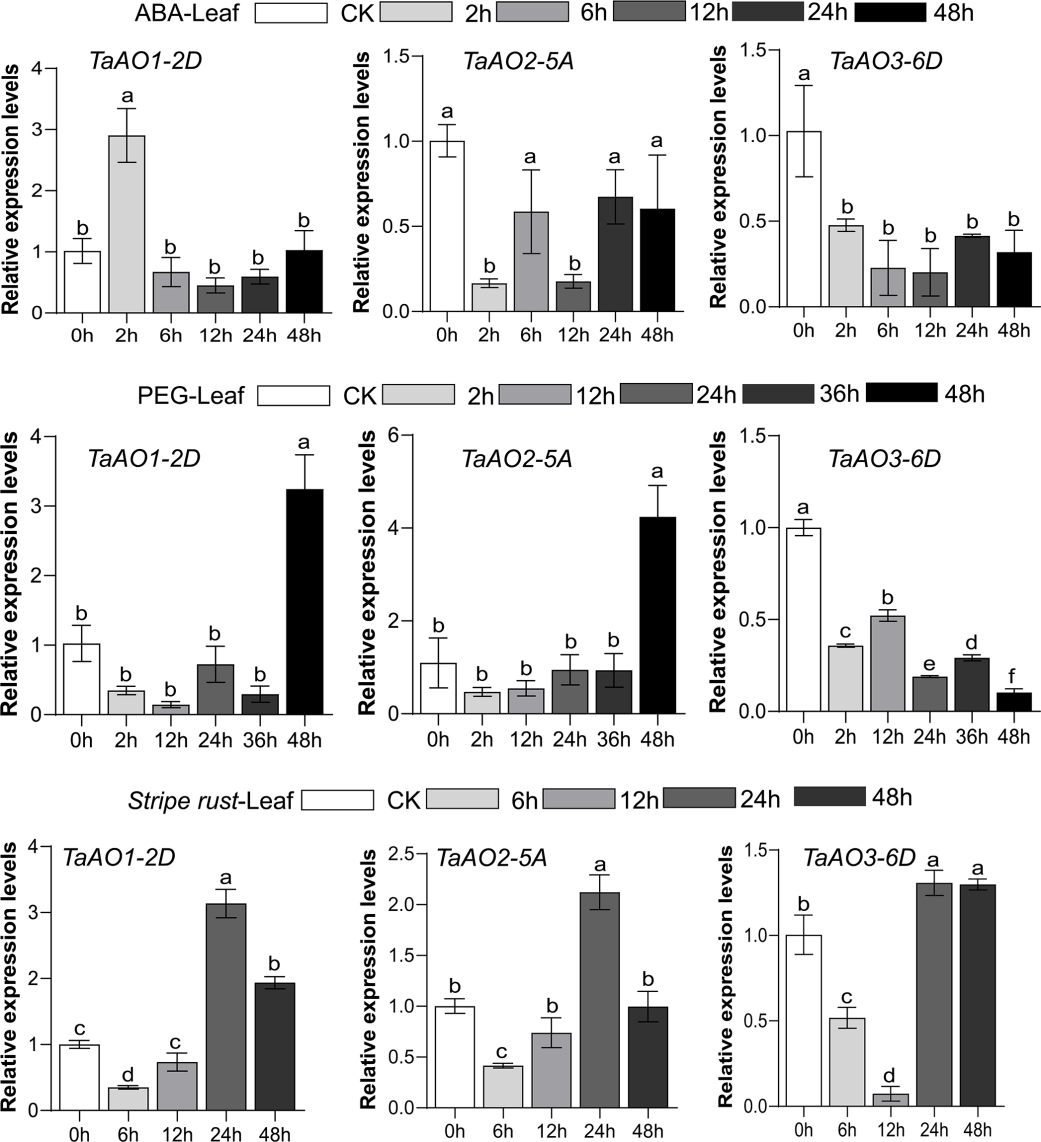
Expression levels of three *TaAO* genes in wheat leaves under exposure to abiotic stress (PEG), plant hormone treatment (ABA treatment), and biological stress (stripe rust infection), as indicated by qRT-PCR. The *x* axis represents time point, and the *y* axis represents expression level. Data from three independent replicates were analyzed; error bars represent the standard deviation. Lowercase letters (a-f) on the bars indicate significant differences determined via one-way ANOVA (P <.05). Plots created using GraphPad Prism 5.

After PEG treatment (**Figure 9**), the expression levels of *TaAO1-2D* and *TaAO2-5A* were decreased for 36 hours, but they were subsequently increased at 48 hours after treatment. In contrast, the expression levels of *TaAO3-6D* were decreased for 48 hours following treatment. In the case of ABA treatment, similar expression patterns were detected in *TaAO2-5A* and *TaAO3-6D*, expression of which was suppressed for 48 hours following ABA treatment. The pattern of expression of *TaAO1-2D* differed slightly from that of the two aforementioned genes: an increase in the expression level of *TaAO1-2D* was observed 2 hours after ABA treatment.

Finally, in wheat inoculated with stripe rust, the general tendency of expression levels of *TaAO1-2D*, *TaAO2-5A*,and *TaAO3-6D* was to increase after an initial decrease. The lowest level of expression of *TaAO3-6D* was observed at 12 hpi, but the lowest levels for *TaAO1-2D* and *TaAO2-5A* were observed at 6 hpi. Compared with the expression level at 24 hpi, the expression levels of *TaAO1-2D* and *TaAO2-5A* were decreased at 48 hpi, while the expression level of *TaAO3-6D* at 48 hpi was roughly equal to that at 24 hpi.

## 4 Discussion

NAD^+^ is an essential cofactor in energy metabolism and electron transfer. Additionally, several reports suggest that NAD^+^ may be involved in plant defense responses (Dutilleul et al., 2003; Zhang and Mou, 2009; Djebbar et al., 2012; Petriacq et al., 2012). Therefore, as the first enzyme of NAD^+^ biosynthesis, AO may influence energy metabolism and plant defense by regulating NAD^+^ content. However, this enzyme has previously only been reported in *Arabidopsis* and maize (Macho et al., 2012; Dong, 2019). In this study, we first conducted a systematic analysis of *AO*-family genes in wheat. The gene architectures, gene duplication events, chromosomal distributions, cis-elements in promoter regions, and expression patterns of *AO* genes in wheat were then further analyzed.

In this study, nine *AOs* were identified in wheat. Compared with other plants, the wheat genome encodes many more *AOs*, which may be due to the heterologous hexaploidy of the wheat genome. In this paper, homology analyses of wheat and three sub-genome donor species were carried out, as a result of which 44 homologous gene pairs were identified, accounting for 90.56% of the wheat orthologous gene pairs. These findings suggest that *TaAOs* are derived from three sub-genome donor species of wheat. Phylogenetic tree analysis of maize, *Arabidopsis*, rice, *T. aestivum*, and its ancestor species (*Ae. tauschii*, *T. dicoccoides*, and *T. urartu*) revealed that Group II only contained *T. aestivum* and its ancestor species. The expression of *AOs* in Group II was high during growth and development, and under biotic and abiotic stress (**Figure 8**), indicating that *AOs* of Group II play an important role in wheat, and that this group has been conserved in the evolution of wheat.

Although AOs are thought to be widely distributed in plants, there has been little investigation of the function of AOs in plants. Up to this point, only two reports on the function of plant AOs have been published (Macho et al., 2012; Dong, 2019). In one of these studies, a differentially expressed *ZmAO* gene involved in energy metabolism was screened from maize CMS-C sterile line C48-2. Compared with the control maintenance line 48-2, *ZmAO* was found to be significantly downregulated in C48-2 during the mononuclear stage of anther development. The *ZmAO* gene may be a positive energy regulator involved in plant growth and development through the NAD^+^ synthesis pathway (Dong, 2019). Furthermore, AtAO2 of *Arabidopsis* is thought to participate in PTI and in resistance to *Pst* DC3000. The expression of *AO* has been found to be increased in non-virulent DC3000-inoculated *Arabidopsis* (Petriacq et al., 2012). Compared with the wild-type, flg22-triggered ROS bursts are significantly suppressed in *AtAO2* mutants, and *AtAO2* mutants are more susceptible to *Pst* DC3000 (Macho et al., 2012). These two *AOs* were both found to be members of Group III in the present study and are localized to chloroplasts (Katoh et al., 2006; Dong, 2019). Among the *TaAOs* identified in this study, *TaAO3-6A*, *TaAO3-6B*, and *TaAO3-6D* were also found to be clustered into Group III, and also located in chloroplasts, which suggests that *TaAO3-6A*, *TaAO3-6B*, and *TaAO3-6D* may have similar functions to those of the *AOs* reported in *Arabidopsis* and maize. Additionally, transcriptome analysis showed that the expression levels of *TaAO3-6A*, *TaAO3-6B*, and *TaAO3-6D* in flg22-treated wheat were increased by 33% to 53% compared with CK. This suggests that *TaAO3-6A*, *TaAO3-6B*, and *TaAO3-6D* may also play a role in PTI in wheat. Moreover, in our study, we found that the expression levels of *TaAOs* belonging to Group III were decreased in wheat treated with powdery mildew and increased in wheat treated with *F. graminearum* (**Figure 8**). As we know, the pathogen of wheat powdery mildew is a biotrophic parasite (Spanu et al., 2010), while *F. graminearum* is a hemi-biotrophic pathogen (Ma et al., 2020). These results suggest that TaAO may play opposing roles in the pathogenesis of hemi-biotrophic and biotrophic pathogens.

Previous studies have revealed that AO plays important roles in biotic stresses and plant development. In this study, we also found that AO may work in response to abiotic stress. The expression of most *TaAOs* was significantly upregulated under exposure to cold stress and downregulated under combined drought and heat stress. This indicates that *AOs* play an important role in the adaptation of plants to cold, heat, and drought stress. In summary, this research lays a foundation for further investigation of the function of *TaAOs*.

## 5 Conclusions

In this study, we systematically identified *AO* genes in wheat genomes. A total of nine *TaAO* genes were identified, which were distributed on three chromosomes of three sub-genomes. TaAOs were clustered into three groups. Gene structure and conserved motifs were similar within each group, but differed among the groups. Transcriptome analysis and real-time PCR assay indicated that TaAOs belonging to Group II were highly expressed in all tissues. *TaAOs* of Group III were found to be involved in PTI response and in the response to ABA treatment, and were found to play a positive role in wheat resistance to *F. graminearum* infection. Furthermore, *TaAOs* might positively regulate the response to cold treatment. These results provide systematic information on AO in wheat and lay a foundation for further research on the functions of *TaAOs.*

## Data availability statement

The original contributions presented in the study are included in the article/**Supplementary Material**. Further inquiries can be directed to the corresponding authors.

## Author contributions

WC, ZF, and WW designed the experiments and directed the writing of the manuscript. YF, MT, and JX performed the experiments and wrote the first draft. YF, PL, LW, and WC revised the manuscript. YF and MT contributed to the data analysis. All authors contributed to the article and approved the submitted version.
